# Using morbidity and income data to forecast the variation of growth and employment in the oral healthcare sector

**DOI:** 10.1186/s13561-016-0088-4

**Published:** 2016-03-19

**Authors:** Dennis A. Ostwald, David Klingenberger

**Affiliations:** 1 WifOR Wirtschaftsforschung, Rheinstr. 22, 64283 Darmstadt, Germany; 2 Institut der Deutschen Zahnärzte (IDZ), Universitätsstr. 73, 50931 Köln, Germany

**Keywords:** Oral healthcare sector growth, Oral morbidity, Growth forecast model, Health satellite account, Macroeconomic model, I11 (analysis of healthcare market)

## Abstract

The perception of the health sector from an economic policy point of view is changing. In the past, health expenditure was mostly seen as a “cost” item, probably because many medical treatments are covered by public health insurance. However, policymakers are increasingly realizing that a growing health sector may be quite beneficial for an economy. It creates employment opportunities and it is relatively resistant to the fluctuations of the business cycle. Input–output analysis could be a useful tool to study the structural change resulting from the growth of the health sector. This paper quantifies for the first time the economic significance of the oral healthcare sector as a component of the German healthcare sector as a whole. The current data for the healthcare sector comes from Health Satellite Accounts, which while comprehensive do fail to answer important questions due to not incorporating certain sectors such as the oral healthcare sector. Therefore on the basis of the Health Satellite Account a specific Satellite Account for the oral healthcare sector is created by using billing data as well as epidemiological data, provided by several dental associations and the Institute of German Dentists. Based on this added information, gross value added data and the number of employees in the oral healthcare sector are computed. Gross value added in 2010 amounted to €13.4 billion, with around €4 billion being attributable to the secondary oral healthcare market; the market for solely out-of-pocket payments. In a second step the paper develops a model to forecast oral healthcare sector growth based on various explanatory variables such as demographic change, take-up behaviour, medical-technical progress, oral morbidity, aggregated supply (collective dental treatment times) as well as income levels and distribution, where the latter two are considered to be of particular importance. According to this model, by 2030 gross value added in the oral healthcare sector will amount to €15.9 million, which corresponds to a 19.2 % increase. The secondary oral healthcare market will be the key to this increase since the model predicts a disproportionately high growth of 60.3 % bringing the total to €6.3 million gross value added in 2030.

## Background

In many countries, including Germany, population ageing goes hand in hand with growing per-capita incomes. These two trends generate a situation in which the demand for all kinds of health products is rising [23]. In economic terms, this induces structural change in the composition of private consumption expenditure as well as public spending. The perception of the health sector from an economic policy point of view is changing. In the past, health expenditure was mostly seen as a “cost” item, probably because many medical treatments are covered by public health insurance. However, policymakers are increasingly realizing that a growing health sector may be quite beneficial for an economy. It creates employment opportunities and it is largely immune to the fluctuations of the business cycle [22]. Therefore this paper focusses on the oral healthcare sector, which is an important component of the healthcare sector, which itself is one of the strongest parts of the German economy in terms of volume of sales and employment [19]. The Health Satellite Account (HSA; in German: GSK) which was previously developed, on behalf of the Federal Ministry of Economics and Technology (BMWi) presents for the first time the healthcare sector in accordance with the categories used in the National Accounts (NAs; German: VGR) [10, 11, 29]. The HSA constitutes a robust statistical basis that permits a comparison of the healthcare sector with the economy as a whole or with other sectors. For instance, it showed that in 2009 the German healthcare sector accounted for some 10.7 % of the macroeconomic gross value added. In the same year approximately 14.2 % of the active population was employed in this sector. In addition, 7.3 % of all exports of goods and services were attributable to enterprises in the healthcare sector. Given the pace of medical-technical progress and ongoing demographic changes, above-average growth and employment may be expected in the healthcare sector [26, 32].

The HSA was established based on official healthcare sector statistics with a view to quantifying the significance of growth and employment in that sector. The figures which are considered include data from the National Accounts (NAs) and the Federal Health Monitoring System (GBE), as well as various specialized statistics published by the Federal Statistical Office. The current body of information on the healthcare sector, which while comprehensive, fails to answer some important questions; for example the significance of growth and employment in the oral healthcare sector, which unlike the healthcare sector as a whole cannot be incorporated as such in NA categories. To operationalize the published HSA figures these results were combined with and matched to billing data from the KZBV (National Association of Statutory Health Insurance Dentists), the “Bundeszahnärztekammer” (German Dental Association) as well as with epidemiological data from the Institute of German Dentists (IDZ).

As a result– based on a specific Satellite account for the oral healthcare sector –the study quantifies for the first time the significance of the oral healthcare sector as a component of the German healthcare sector. The baseline year of this model is 1996, because the required extensive billing data was only available from this point.

This contribution starts with a brief description of the key features of the German (oral) healthcare system. In the second step it sets out the conceptual framework to measure the growth and employment effects of the oral healthcare sector with a Satellite Account within the System of National accounting (SNA). Last but not least a methodology that can be used to forecast the growth and employment effects of the oral healthcare sector is discussed. Therefore the models’ variables, assumptions and the underlying data sources are described. A brief outline of the principal results of the forecasting model is then given. The paper concludes with a discussion of the validity and limitations of the model.

### Organisation and financing of the German (oral) healthcare system

The German healthcare system can be characterised as a statutory social insurance model, in other words the organisation and financing of the healthcare system is based on the traditional principles of solidarity, insurance, and self-administration. The role of the federal government is limited to setting the statutory framework for healthcare.

Employees whose monthly salary is below the limit for mandatory insurance (2015: 4575 euro) are compulsory members of the statutory health insurance (Gesetzliche Krankenversicherung [GKV]). A salary above the limit for mandatory insurance gives the employee the option of becoming a member of a private health insurance company (Private Krankenversicherung [PKV]). In 2015, approximately 86 % of the population were insured with statutory health insurance, and 11 % of the population were fully insured by a private health insurance provider. In addition, there are further special systems of social protection in the form of gratifications (for civil services), free medical care (for law enforcement officials and civilian servants among others), and free healthcare provision (for soldiers).

There is a uniform contribution rate to finance the statutory health insurance which currently is 15.5 %, from which the employer pays 7.3 % and the employee pays 8.2 % of the earned income [20].

In Germany, health policy is organised as a contribution (or revenue) related expenditure policy. One main policy goal is to avoid an increase in the contribution rate of the statutory health insurance. Furthermore, the revenues are dependent on the structure of the labour force as most employees are compulsory insured in the statutory health insurance. The macroeconomic trend of decreasing labour income shares has proved to be particularly problematic. As a consequence the financial basis of the statutory health insurance is eroding and co-payments by the patients are becoming more and more common.

Within the scope of standard care, those with statutory health insurance receive the necessary dental services as transfers in kind; from which there are special regulations for the reimbursement of costs. However, for the privately insured the cost reimbursement principle and the principle of contractual freedom apply, in other words the scope of the insurance chosen by the insured party and their individual risk of illness determine the level of their contributions. On the one hand, patients can freely choose their doctor. On the other hand, people have to go to a dentist once a year otherwise they lose entitlements for benefits. The starting point of the study will be based on the changing circumstances in the financing of healthcare within Germany which will be used as a basis for forecasting future sales and employment in the oral healthcare sector until 2030.

## Methods

The following sections describe the individual variables and assumptions of the oral healthcare sector quantification model, commencing with an introduction to the specific features and limits of the HSA and its potential use as a basic data resource as well as a forecasting model of the oral healthcare sector.

### Establishment of a Satellite Account in accordance with the example of the healthcare sector

Due to the increasing importance of the healthcare sector, the HSA was developed under a research project conducted on behalf of the Federal Ministry of Economics and Technology (BMWi) with a view to analysing the overall effects of that sector based on the National Accounts (NAs). The Satellite Account for the healthcare sector in Germany was established in 2009 [10, 11,] and has been developed every year [1, 13, 29].

The HSA was developed because the Federal Statistical Office’s regular publication of its Health Expenditure Account (GAR) only showed final demand in Germany, but not the significance of the healthcare sector in terms of growth and employment, given that the sector is increasingly funded by households directly.

The HSA comprised of setting out the definitions of the various segments of the healthcare sector. The organisation of the healthcare sector is generally far from being a pure “market”, since prices are negotiated between institutions and legislated by so-called remuneration agreements. In this respect price fluctuations do not follow the laws of supply and demand. *Herder-Dorneich* describes the German healthcare system as a “quasi-market system” in which limited medical and technical competition is allowed for statutory and private (reimbursement) insurances [12]. Therefore in this paper the authors distinguish between the primary oral healthcare *sector* and the secondary oral healthcare *market*.

In the HSA the healthcare sector was divided into groups of accounts, such as production accounts (including a derived workforce account) and input–output (I/O) tables of the NAs. These accounts break down the healthcare sector by supply, demand and distribution. Due to this it is possible to show the production values, gross value added, intermediate inputs, exports and imports, and number of workers for each production segment of the healthcare sector.

The development of the HSA thus provides a firm definition of the healthcare sector as a basis for statistical analysis of its macroeconomic significance. Furthermore, the HSA for the first time identifies and describes the healthcare sector’s contribution to growth and employment and its economic interdependencies within the economy as a whole [29].

### Creating an Satellite Account for the oral healthcare sector

Satellite analysis have earned growing interest in research throughout the recent years. As the common national accounts are able to give quite a good overview of the main key data concerning the economy in consideration and about conceptual interconnectedness between the existing sectors, Satellite Accounts enable to answer more detailed questions about single areas of interest or – to be more specific - overlapping sectors of the economy.

Single areas of interest in terms of their economic performance and the impulses erupting from it, which are of political concern, are most often not pictured in National Accounts. The goal of Satellite Accounts is thus to picture the performance of cross-sectorial branches in the economy, which are of political concern. In order to illustrate the fact, that Satellite Accounts are already state of the art in the field of input–output-calculations, the following definition for Satellite Accounts is taken from SNA (1993) [33, 34]:

‘Satellite Accounts or Systems generally stress the need to expand the analytical capacity of national accounting for selected areas of social concern in a flexible manner, without overburdening or disrupting the central system.’

The aim of this publication is to use morbidity and income data to forecast the variation of growth and employment based on a specific Satellite Account for the oral healthcare sector. The analysis of the growth and employment effects of the oral healthcare sector in the same way as the HSA is not possible. This is because the goods and services specific to the oral healthcare sector cannot be distinguished clearly within the current classification used to define the healthcare sector. The reason is that the oral healthcare sector is located in different classifications of the healthcare sector. As an example the dental industry is part of the medical-technological goods. Hence the HSA methodology cannot simply be transposed to the oral healthcare sector without the need for certain intermediate calculations. The calculations would lead to the disaggregation of the statistical classifications to the needs of the oral healthcare sector.

To portray the significance of the oral healthcare sector in terms of growth and employment, a calculation model for quantification of the sector was developed. For this purpose the goods and services of the oral healthcare sector were disaggregated with the help of several primary statistics. The primary data included billing data from the KZBV [National Association of Statutory Health Insurance Dentists] (statistics on individual treatment items/BEMA^1^), the results of the GOZ^2^ analysis carried out by the “Bundeszahnärztekammer” [German Dental Association] and the KZBV, and the basic epidemiological data for the oral healthcare sector (IDZ Oral Health Studies DMS I to IV) [25].

### Segmentation of the oral healthcare sector

With the aid of additional data, the individual segments of the oral healthcare sector – e.g. dental practices, dental technology, dental supplies, conservative dentistry, prosthetic dentistry, etc. – can be identified and categorized by sales and expenditure categories. This systematization is necessary to permit differentiated analyses, with an example being different rates of variation in individual segments. Next, a *first stage* definition of the oral healthcare sector is conducted in accordance with the approach used to establish a Satellite Account.

**Fig. 1:**
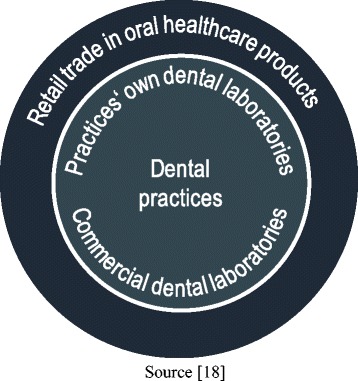
Layered model of the oral healthcare sector

As with the layered model of the healthcare sector [14, 27], the oral healthcare sector is divided into three layers (see Fig. 1):

» The *first layer* – the core of the model – comprises the *dental-practice segment*. It includes all treatments carried out directly on patients at outpatient dental practices.
» The *second layer* is the *dental-technology segment*. This is deemed to include both practices’ own laboratories and commercial dental laboratories.
» Finally, the *third layer* relates to the *retail trade in oral health products* – i.e. dental and oral care products as broadly defined [18].

In addition to the breakdown of the oral healthcare sector by segments, analysis on the basis of goods and funding is possible. The four-field breakdown of the oral healthcare sector is shown in Table 1 below [11, 29].

**Table 1:** Four-field breakdown of the oral healthcare sector

	Breakdown by funding	
	First Oral Healthcare Market	Second Oral Healthcare Market
Breakdown by goods	Private or statutory health insurance schemes (comprehensive insurance or state funds)	Private funding (consumer spending)
Oral healthcare sector core segment	e.g. standard treatment in dental and orthodontic practices (“need dentistry”)	e.g. professional tooth cleaning (PZR); orthodontic treatment for adults (“want dentistry”)
Goods as defined in the Health Expenditure Account (GAR)		
Oral healthcare sector extended segment	e.g. necessary bite correction appliances	e.g. tooth bleaching, veneers, toothpastes, dental floss, manual and electric toothbrushes
“New” oral-health-related goods (subjective purchase decision)		

Source: Own diagram based on Henke et al. [11, 28]

With the four-field breakdown shown above, goods and services/treatments can be divided, firstly, into a core segment and an extended segment. The *core segment of the oral healthcare sector* comprises goods as defined in the Health Expenditure Account. This primarily includes treatments provided by dental practices under the heading of standard care. The *extended segment of the oral healthcare sector* also includes “new” oral-health-related goods purchased on the basis of a subjective decision (a request for a given type of care).

Secondly, in accordance with this approach, goods and services/treatments can also be distinguished by their funding and are assigned to either the primary oral healthcare sector or the secondary oral healthcare market. Whereas goods and services/treatments belonging to the *primary oral healthcare sector* are funded primarily by the statutory health insurance scheme (GKV), private health insurance (PKV) or public funds – that is, from contributions and taxes – the *secondary oral healthcare market* is composed solely of direct consumer spending by households in the form of out-of-pocket payments. The distinction has been chosen for methodological reasons, from which the main reason is that the same distinction is used in the HSA. In fact, many standard treatments are co-financed by insurances and households. Statistically speaking, both sectors are related to each other and the sales trend in both sectors shows some evidence for a complementary relation.

The services offered on the *secondary oral healthcare market* are part of the “want dentistry”, they did not fall under the category of individual health services (IGEL-Leistungen), because the patient only has to pay the additional costs beyond standard care, but not the costs as a whole.

In the second stage, the oral healthcare sector was quantified on the basis of the available primary and secondary data. In this paper, the dental-practice segment – i.e. the core segment of the layered model, was first quantified by the Federal Health Monitoring System (GBE), which included the Healthcare Expenditure Account, billing data from the KZBV, the data sets of the GOZ analysis and the data held by the IDZ [18].

In this research approach, the calculations concerning the HSA in Germany have been disaggregated by using the primary statistics of the oral healthcare sector. Stating that based on the treatment items billed in accordance with the BEMA and GOZ, the determination of the annual volume of sales in the dental sector and the structure of those sales was generated and implemented in the methodological approach of the HSA. Secondly, the figures for individual treatment items together with the GOZ analysis permit in-depth secondary statistical evaluations. To summarize, in this approach we used primary statistics of the oral healthcare sector to locate the goods and services within the HSA to calculate impact of the oral healthcare sector to growth and employment to the German GDP. To improve our calculation we used statistics of dental registrations and empirically proved time requirements estimates for dental care to forecast the supply side in a proper way [24]. In addition, the forecast of value added and employment figures are based on assumptions regarding the variation of oral morbidity, for example the DMS IV Oral Health Study.

### First results of the Satellite Account for the oral healthcare sector as the basis of a macroeconomic forecast model

The data sources outlined above can be used to calculate the volume of sales in the dental-practice segment of the oral healthcare sector, which can be broken down by treatment types for the period 1996 to 2008. The table below shows the breakdown of the individual dental-practice segment subgroups to be analysed, together with the corresponding treatment types where data is available. (Table 2).

**Table 2:** Group classification of dental-practice treatment types

Group	Treatment types
Conservative dentistry segment (including surgery)	Subgroups: conservative treatments (KONS) and periodontology (PAR): • BEMA: o General, conservative and surgical treatments o Periodontal treatments (excluding jaw fracture) • GOZ: o General dental treatments o Conservative treatments (excluding crowns) o Surgical treatments o Treatments for pathology of the oral mucosa and periodontium
Prosthetic dentistry segment	Subgroup: Prosthetics: • BEMA: o Prosthetics • GOZ o Prosthetic treatments o Crowns (from “Conservative treatments”)
Other segments	Subgroup: Other: • BEMA o Orthodontics • GOZ o Prophylaxis o Orthodontic treatments o Fitting of occlusal appliances and bite guards o Functional analysis and therapy o Implantology treatments • Scale of Fees for Medical Practitioners (GOÄ)

Source: Klingenberger et al. [18]

There is a significant difference between the dental-practice segment (layer 1) and the dental-technology segment (layer 2). While patients account directly for the entire range of treatments and services provided by the dental-practice segment, the role of dental technology is confined to supporting dental-practice activity in individual classes of treatment, such as prosthetics, implantology or orthodontics. Hence the demand for dental-technology goods and services is determined by the volume of dental-practice activity in these fields. For this reason, the main focus of the model is on forecasting the growth in the dental-practice segment. Finally, statistics on the variation of sales of oral health products were used to estimate the volume of sales in layer 3 [11, 16].

The growth of the oral healthcare sector is deemed to be represented by the *gross value added* actually achieved. Gross value added is composed of the volume of sales or expenditure, less intermediate inputs, the growth of value added being determined in accordance with actual resource allocation and actual variation of productivity [9].

By using sales and expenditure categories for the oral healthcare sector, the macroeconomic parameters of “gross value added” and “intermediate inputs” are derived from the Health Satellite Account. The intermediate-input ratios applicable to comparable goods from the HSA are used for this purpose. The number of workers is calculated in the same way, using goods-specific productivities of labour from the HSA as auxiliary variables (proxies). A second step comprises of validation, by comparing the resulting *numbers of workers*^3^ in the oral healthcare sector by means of the Federal Statistical Office’s Healthcare Labour Force Account (GPR).

This approach permits detailed analysis of the oral healthcare sector. In addition, the oral healthcare sector can be analysed as shown in Table 1. The four-field breakdown of the oral healthcare Sector illustrates the possibility to analyse the sector in terms of both goods and type of funding the same way the HSA does.

Table 3 illustrates the variation of gross value added and numbers of workers for each layer of the oral healthcare sector in 2010.

**Table 3:** Gross value added and numbers of workers in the oral healthcare sector in 2010

Oral healthcare sector	Gross value added (million €)	Workers (thousands)
	2010	2010
Layer 1	9 076.84	382.20
*of which 2nd OHM*	*2 232.90*	
Layer 2	3 408.04	
*of which 2nd OHM*	*838.38*	
Layer 3	890.75	27.36
*of which 2nd OHM*	*890.75*	
Total	13 375.63	409.56
*of which 2nd OHM*	*3 962.03*	

Source: own figure based on [19]

### Development of a forecasting model on basis of the Satellite Account

The forecasting model is based on the specific Satellite Account for the oral healthcare sector used to quantify the impact of the oral healthcare sector in the Future. The extension is based on using additional primary data besides macroeconomic indicators. The future growth and employment effects of the oral healthcare sector depend on a number of parameters that form the conceptual framework of the model. The following description begins with an outline of the explanatory (statistically independent) variables of the regression model. These variables have a determining effect on the (statistically dependent) variables of the regression model that are to be explained.

### Determinants of growth – explanatory variables

For the purposes of the forecasting model, the following explanatory variables for growth in the oral healthcare sector were identified:

» oral morbidity
» demographic change [2, 3],
» changes in demand [12],
» income levels and distribution [24], and
» medical-technical progress [4].

*Morbidity* describes the incidence of a specified pathology in a given group of subjects over a defined period. Morbidity incidence increases with age, resulting in a greater demand for healthcare services later in life [2, 7].

Therefore the ageing of society is subsumed in the concept of *demographic change* results in additional demand. Ageing is attributable to increased life expectancy coupled with a low or gradually falling birth rate. The number of older people will decrease over the long run, but within the year 2030 the number of the cohort between 65 and 80 years old people (the babyboomer generation) will increase [4]. As a result the “demand” impact of this generation will influence the oral healthcare sector within this period of time significantly [8, 35, 36]. Other growth factors come from an increasing awareness of health issues and consequent changes in the population’s *demand* for services. The significant increase in dental awareness was observed in recent times is a particular stimulant of growth. The rising demand for example for aesthetic dentistry bodes well for growth of the second oral healthcare market in particular.

Another vital parameter for the oral healthcare sector in this connection is *income*. In an ageing society the retirement pensions are becoming more and more important, hence this is having an impact on income distribution [30]. To summarize not only the level of income but also its variance is important to forecast the impact of the oral healthcare sector.

The extent of *medical-technical progress* is as a rule assessed by the proportion of the total volume of sales accounted for by new or improved products and techniques, R&D expenditure or the number of patent applications. In the context of modelling, technical progress can ultimately be assessed only in accordance with the variation of labour productivities [26].

The following sections address in more detail the two principal parameters, or explanatory variables; morbidity and income, and demonstrate their effect on the variation of growth in the oral healthcare sector. It is necessary to mention, that only two explanatory variables are described in detail. Our methodical approach consists of extended time series-analysis to estimate a status quo forecast. By using an extended time series for the supply and demand side, the model considers changes in the oral health care system over the last couple of years, like structure of expenditures, revenues or the institutional and socio-economic setting. As example the increase of expenditures because of the increasing number of dentists as a sign of supply induced demand is considered.

### Morbidity and forecasting oral morbidity

Oral morbidity is the occasion for activity in the dental-practice (layer 1) and dental-technology sectors (layer 2). The dental industry is made up of the “latent treatment requirement” which is defined as a condition that accrues over time thus lowering the health related utility of an individual, although no treatment has yet been given. When a dental treatment is taken up in the professional care system, this constitutes a manifest demand. Hence the latent treatment requirement determines the manifest demand for treatment in the oral healthcare sector.

Oral Morbidity (and the latent treatment requirement) is the reason for activity in the dental-practice or dental-technology sector therefore, assumptions concerning its variation are essential for the purposes of a calculation or forecasting model. With regard to the variation of oral morbidity, the focus is then on three major (epidemiological) clinical pictures of dental caries, periodontitis, and tooth loss [18].

It is also important to keep in mind that the variation of oral morbidity – and hence also the latent treatment requirement and manifest demand. This is crucially determined by changes in demographic structure, because the incidence of oral pathology varies significantly with age [25]. So the ageing of the population has implications for oral health. The continuous trend of “caries decline” has been attributed to preventive care [21]. The growing number of older adults who have retained their natural dentition into advanced old age has created the assumption, that for the case of oral health the “compression of morbidity”-thesis is appropriate [5, 15, 17]. For this reason, information on demographic change must also be included in the consideration of morbidity.

Oral morbidity forecasts are drawn up on using the available data resources integrating them into the model as a primary explanatory variable. The relevant morbidity data was taken from the Fourth German Oral Health Study (DMS IV), a large-scale population-representative cross-sectional study. A total of four population-representative cross-sectional studies with a social-epidemiological research design have been issued for Germany to date. The health surveys, covering a 16-year period, record the core prevalence of caries decline, periodontal pathology and tooth loss, including levels of dental treatment, which were then used for target projections up to 2030. The target projections themselves were generated from a retrospective consideration of the relevant morbidity trends (from 1989/92 to 2005) at national level [18]. The DMS studies also allow for demographic change, by recording morbidity separately for different age cohorts [25].

### Income growth and distribution

In the primary healthcare sector, income substantially influences the funding of the statutory health insurance scheme, and hence the level of insurance benefits offered. Income carries greater significance in the secondary healthcare market, since the healthcare goods and services available on this market are by definition funded privately. Income operates as a consumption-limiting factor, which exerts a decisive influence on the manifest demand for health-related goods and services on the secondary healthcare market [19].

Income is described in two ways; by its per capita variation and by its variance. The effect of the income parameter is accounted for in the model by the construction of the explanatory variable “distribution-adjusted per capita gross domestic product”. The construction of this explanatory variable is based on a mathematical combination of two data series [18].

The *first data series* represents nominal gross domestic product in relation to the relevant population size for the period 1996 to 2030. Average annual growth of per capita nominal gross domestic product (GDP) is recorded for the period 1996 to 2009 and extrapolated conservatively to 2030 for forecasting purposes. Next, 1996 is taken again as the baseline year for the data series (index = 1). Hence the first data series represents the long-term variance of per capita nominal gross domestic product, with a baseline year of 1996 [18].

The *second data series* represents the variation of unequal distribution of incomes for the period 1996 to 2030. A suitable parameter for this purpose is the Gini coefficient (GC) applied to net household incomes. In the case of a completely equal distribution of income the GC assumes a value of 0, whereas its value is 1 when the distribution of incomes is completely unequal. Historical data availability is excellent, so that the long-term variation of the measure of distribution can be derived from a time series consideration [6, 31]. However, the inverse Gini coefficient (IGC) is better suited for the purpose of combining the two data series that represent the incomes parameter. The IGC is obtained by subtracting the Gini coefficient from 1 for each historical data point. If the Gini coefficient increases with time – indicating increasingly unequal income distribution – the IGC decreases correspondingly. To ensure consistency between the two data series, a baseline value of 1 in 1996 is assumed for the IGC. The long-term variation of the IGC as thus defined is established for the period 1996 to 2009 using a linear regression calculation and extrapolating it up to 2030 for forecasting purposes. The second data series thus represents the long-term variation of the IGC, with a baseline year of 1996 [18]. Unfortunately the data consistency before 1996 is not given, due to the fact that there have been changes in reporting the data.

The income parameters are derived by linking the two data series described above, thus allowing the explanatory variable “distribution-adjusted per capita gross domestic product” to be calculated. Finally, the percentage variations of this variable referred to the baseline year 1996 are incorporated in the model [18]. The result is shown in Table 4.

**Table 4:** Rates of change of distribution-adjusted gross domestic product per capita

Year	Rate of change	Year	Rate of change	Year	Rate of change	Year	Rate of change	Year	Rate of change
1996	0.0000	2003	0.1246	2010	0.2637	2017	0.4213	2024	0.5981
1997	0.0165	2004	0.1464	2011	0.2851	2018	0.4454	2025	0.6250
1998	0.0406	2005	0.1602	2012	0.3069	2019	0.4698	2026	0.6524
1999	0.0619	2006	0.2014	2013	0.3291	2020	0.4946	2027	0.6802
2000	0.0845	2007	0.2531	2014	0.3516	2021	0.5198	2028	0.7085
2001	0.1063	2008	0.2868	2015	0.3744	2022	0.5455	2029	0.7372
2002	0.1172	2009	0.2426	2010	0.2637	2023	0.5716	2030	0.7664

Source: own calculations

### Forecasting the growth and employment effects as a three-stage process

The aim here is to forecast the volume of sales in the oral healthcare sector for the period 2009 to 2030 by applying a regression calculation. Using the historical data for the segments mentioned above, *forecasting of the oral healthcare sector* is conceived as a three-stage process. The variation of the dental-practice segment (layer 1) is forecasted first, subsequently the variation of the dental-technology segment (layer 2) is calculated. Then the retail trade in oral care products (layer 3) is considered. The procedure for calculating the volume of sales by means of explanatory variables is presented below.

Extensive billing data for the period 1996 to 2008 is available for the *dental-practice segment of the oral healthcare sector* (layer 1). The billing data is used to approximate the volume of sales in the dental-practice segment). So the effect of the manifest demand for oral healthcare services can be represented using the regression model (see Table 4).

The volume of sales in the *dental-technology segment* (layer 2) depends indirectly on the manifest demand for oral healthcare services in the dental-practice segment (see Table 4). For this reason, the correlation between the volume of sales in the dental-practice segment and that of the dental-technology segment enumerated is determined for the period 1996 to 2008 on the basis of a regression calculation. The annual volume of sales generated in the dental-technology segment of the oral healthcare sector is represented as an aggregate by means of a single variable to be explained.

Forecasting of the variation of the segment *retail trade in oral health products* (layer 3) is limited by the lack of an extended data series. However, the available data shows that in terms of volume of sales dental and oral care products, stand at some 1.3 billion euro per annum (in 2008 and 2009), and are the fourth largest group in the personal care products category [16]. In addition, sales of oral care appliances are found to have grown constantly in the past few years, to about €150 million [37].

The next step is forecasting of the variation of the volume of sales in the oral healthcare sector up to 2030 based on the variation observed in the past (of the actual correlation between manifest demand and volume of sales). Lastly, future growth of gross value added is calculated from the forecast volume of sales using segment-specific intermediate-input ratios [18]. The segment-specific intermediate-input ratios applied are determined from the value-added approach as to Ostwald and Ranscht [27] and from existing HSA publications [11, 29]. The future variation of the Employment rate is established from the labour productivities specific to economic or production sectors. Using a similar methodology to that of the “Sachverständigenrat” (Council of Experts on Healthcare) [31], sector-specific labour productivities are applied, in this case again determined from the value-added approach used by Ostwald and Ranscht [27] and from published HSA material [11, 29]. The specific labour productivities^4^ are then linked to the numbers of workers from past years to permit determination of the employment effects accrued from the value added [19].

## Results

On the basis of the calculations and forecasting model described, a brief overview of the main results is given below.

To allow for uncertainty in the forecasting of the future income variance, the variation of sales is presented in three different income scenarios for the primary oral health care sector and the secondary oral healthcare market respectively. The baseline scenario assumes an annual income growth of 1.96 %, which corresponds to average income growth in the period 1996 to 2008. In addition, the effect of smaller annual growth (1 %) (lower scenario) and of greater annual growth (3 %) (upper scenario) on sales in the primary oral healthcare sector and the secondary oral healthcare market is predicted.

In the baseline scenario, the forecasted volume of sales for the oral healthcare sector as a whole in 2030 is €22.71 billion. This represents an increase over 2010 of €4.3 billion, or 19.0 %, the secondary oral healthcare market being expected to account for a higher proportion of total sales in 2030. Whereas the privately funded segment amounted to 29.6 % of the oral healthcare sector’s volume of sales in 2010, by 2030 this proportion increases to 39.8 %. The secondary oral healthcare market will increase from €6.73 billion in 2010 to €10.76 billion in 2030 (a relative increase of some 60 %). This means that the growth of sales on the secondary oral healthcare market (2^nd^ OHM) is forecast to be significantly higher than that of the total volume of sales in the oral healthcare sector as a whole (19.0 %), thus emphasizing the growing importance of private forms of funding in the oral healthcare sector. Furthermore, throughout the scenarios the secondary oral healthcare market is seen to be expanding significantly. The primary oral healthcare sector, on the other hand, will exhibit more differentiated performance in accordance with the variation of incomes.

The variation of employment similarly underlines the importance of the oral healthcare sector in labour market terms. Starting from a figure of 410 000 in 2010, the number of workers in the oral healthcare sector is forecast to increase to approximately 486 000 in 2030. This corresponds to an 18.6 % increase in employment in the period 2010 to 2030. This contrasts to the trend of employment in the economy as a whole, which is expected to show negative growth (−0.6 %) up to 2030, the oral healthcare sector is likely to experience an annual increases in employment of some 0.86 %. Given stable contextual conditions, dentistry in Germany will contribute to preserving jobs and creating new employment opportunities.

The purpose of the specific Satellite Account for the oral healthcare sector and the additional forecasting model developed here is to measure and estimate the growth and employment effects of the oral healthcare sector in Germany. The methodological approach is based on the research project “Development of a National Health Satellite Account”. For the first time it is possible to estimate the impact of the three layers of the oral healthcare sector on growth and employment in Germany.

The description of the forecasting model adduces two crucial determinants of growth in the oral healthcare sector – namely, morbidity and income. Morbidity and income likewise have a significant impact on the growth of the oral healthcare sector. Statistics of dental registrations and empirically proved time requirements estimates for dental care were used to take the supply side into account.

The first result of the forecasting model shows that the oral healthcare sector will grow during the next years in terms of gross value added and numbers of workers. According to this model, by 2030 gross value added in the oral healthcare sector will amount to €15.9 million, which corresponds to a 19.2 % increase. The secondary oral healthcare market, which is likely to account for nearly 40.0 % of the oral healthcare sector in 2030, will gain considerable importance. For the period under research, the figures for the secondary oral healthcare market reflect the growth of dental awareness in society and the associated control-oriented demand of oral treatments by the patients.

## Discussion and conclusion

The methodology used in this paper is a first attempt to simulate the economic impact of the oral healthcare sector from a macroeconomic point of view. The Satellite Account and the forecasting model are based on the Systems of National Accounting and additional primary statistics. The forecast is a status-quo projection to the year 2030. The variances in some of variables try to describe the influence of the parameters to growth and employment in Germany. However it should be stated that any future healthcare reforms may influence the trends forecasted in the oral healthcare sector (i.e. direction of “cost containment” in the oral healthcare system).

Regarding the demographic change there could be some shifts as well. Especially with new Census’ being conducted and the increasing number of older people within the given period can change the results. Furthermore age itself is a multidimensional factor [8, 35, 36] that needs to be considered in future research. For instance, it would be reasonable to distinguish between the biological age, the psychological age, or the social age. The reason is that each of these dimensions has its own influence on the decision to buy a special product. Therefore, e.g. the linear measurement of age by calendar years could be changed. Additionally it could be distinguished between age-, period- and cohort effects, which it is not possible using cross-sectional data described above. For future research it may be appropriate to include additional data of the supply side (e.g. changing shares of employed and resident dentists, changing forms of practice).

Conclusively the postulated intermediate-input ratios may vary and labour productivities may grow faster than assumed in the model. Moreover assumed prices (fees and material and laboratory costs) may likewise vary. Given that these parameters are subject to dynamic change, the direction of their variation should be monitored constantly. Fine adjustment of the model would be displayed if the parameters exhibit an unexpected pattern of variation. Last but not least the data set should be brought up to date in order to consider the financial crisis’ impact.

The model permits regular, real-time evaluation of the variation of sales, gross value added and employment in the oral healthcare sector. Aligning forecast data with actual conditions, could then form the basis for a valuable health-economics monitoring system in terms of the heuristics of research.

## Footnotes

1 The Standard Assessment Scale for Dental Treatment Items (BEMA) is the basis for billing dental treatments in the German statutory health insurance scheme.

2 The Dental Fee Schedule (GOZ) is a legal instrument that governs charges for dental treatments in Germany.

3 “Workers” (ET) are defined as employees, self-employed persons or assisting family members focused on acquisition. The number of workers in the oral healthcare sector thus includes not only self-employed dentists in private practice and their practice staff, but also persons employed in the dental industry, dental laboratories and (proportionate) the medical and healthcare products and devices trade.

4 On the basis of retrospective observations the productivity of labour is assumed to increase by 1 % per annum.
